# Supplementary material for: The Success and Safety of Endoscopic Retrograde Cholangiopancreatography in Surgically Altered Gastrointestinal Anatomy
Source: Med Sci (Basel). 2025 Feb 11;13(1):18. doi: 10.3390/medsci13010018 (PMC11843866; doi:10.3390/medsci13010018)
Supplement: Supplementary file 1 [file medsci-13-00018-s001.zip › medsci-3453123-supplementary.pdf]

Supplementary Table S1: Success rate for Billroth II anatomy by endoscope utilized.

Anatomy	Enteroscopy Success	Cannulation Success	Intervention Success
**Billroth II (81 patients, 169 ERCPs)**			
Duodenoscope (n=141)	140 (99.3%)	136 (96.5%)	133 (94.3%)
Pediatric colonoscope (n=23)	20 (86.9%)	13 (56.5%)	12 (52.2%)
Adult colonoscope (n=2)	2 (100%)	2 (100%)	2 (100%)
Rotational enteroscope (n=1)	1 (100%)	1 (100%)	1 (100%)
Single-balloon enteroscope (n=2)	1 (50%)	1 (50%)	1 (50%)

Supplementary Table S2: Success rate for classic Whipple anatomy by endoscope utilized.

Anatomy	Enteroscopy Success	Cannulation Success	Intervention Success
**Classic pancreaticoduodenectomy (76 patients, 152 ERCPs)**			
Pediatric colonoscope (n=116)	108 (93.1%)	99 (85.3%)	96 (82.8%)
Adult colonoscope (n=14)	14 (100%)	14 (100%)	14 (100%)
Rotational enteroscope (n=6)	6 (100%)	5 (83.3%)	5 (83.3%)
Single-balloon enteroscope (n=14)	9 (64.3%)	7 (50%)	7 (50%)
Double-balloon enteroscope (n=2)	2 (100%)	2 (100%)	2 (100%)

Supplementary Table S3: Success rate for pylorus-preserving pancreaticoduodenectomy anatomy by endoscope utilized.

Anatomy	Enteroscopy Success	Cannulation Success	Intervention Success
**Pylorus-preserving pancreaticoduodenectomy (14 patients, 22 ERCPs)**	19 (79.2%)	16 (66.7%)	16 (66.7%)
Pediatric colonoscope (n=21)	16 (76.2%)	14 (66.7%)	14 (66.7%)
Single-balloon enteroscope (n=2)	2 (100%)	1 (50%)	1 (50%)
